# 

**Published:** 1971-10

**Authors:** 


### CONTENTS

Compression Plating for Non-union of the Tibia ... ... 67
E. Michelinakis

Problems of Anticoagulant Control—II, Which Test? ... 71

T. J. Hamblin

Wisdom from the Past ... ... ... ... ... ... 74

Obituary ... ... ... ... ... ... . ... 75

Bristol Medico-Chirurgical Society ... ... ... ... 78

South-West Orthopaedic Club ... ... ... ... ... 79

The Medical Library ... ... ... ... ... ... 80

Index to Vol. 86 ... ... ... ... ... ... ... 82

F. Bailey & Son Ltd., Dursley, Glos.

